# Monthly Summary

**Published:** 1874-03

**Authors:** 


### MONTHLY SUMMARY.

---

*Transplantation of Teeth.*—In a former number of this JOURNAL, (April, 1871,) we noticed the fact as established, that teeth are capable of being transplanted so as to retain their vitality, forming new attachments, like grafts on trees. Dr. Isidor L. Lyons, an eminent English dental surgeon, furnishes to the *London Lancet* for November, the result of his experience in the operation. He refers to the two attachments that are severed in extracting a tooth—first, the periosteal adhesion, and second, the nervous and vascular connection. There is no reason, he says, why the alveolo-periosteum should not again unite to the tooth, seeing that if a piece of periosteum be stripped off a bone it will re-unite if placed in contact with the bone and kept at rest. The union of the divided ends of a nerve is also a recognized fact; but, even supposing the latter impossible the tooth would merely be in the condition of one which has had its pulp destroyed, a common operation in dental surgery. Out of twelve cases on which he has operated, nine were successful, and three failures. It is difficult, he says, to induce patients to submit to an operation in regard to which they are so incredulous. The pain pursued by Dr. Lyons, he describes in these words: "A tooth which is to be replanted should be carefully extracted, and as little as possible of the surrounding tissues lacerated; it should then, unless the operation be simply for the destruction of the dental pulp, and when the periosteum is healthy, be immersed in some antiseptic fluid, such as diluted carbolic acid or chloride of zinc, (the latter from experience being preferred;) the socket should then be swabbed out some half-dozen times with a strong solution of the same antiseptic employed. The tooth, if carious, should be plugged and returned to its place. If there is any thickening of periosteum, fibrous growth, sac of abscess, or absorption at the extremity of the fang, it should be excised before replantation. Should patient complain of pain arising from the operation, prescribe poppy fomentations, although the pain is rarely more than what is due to the tenderness of parts from the laceration of soft tissues after the extraction of the tooth."

This process is substantially the same as we described in the *Pacific M. and S. Journal* nearly three years ago, and then credited to another English dentist, M. Coleman, who had succeeded in nine out of fourteen cases. Dr. Lyons makes no reference to him, except to mention that he "suggested" the operation.—*Pacific Med. and Surg. Journal.*

---

*Mental Hygiene.*—But there is still another class of facts differing from any of those mentioned, that has a powerfull influence upon longevity, viz., the influence of mind upon the body. Mental training, a well-balanced mind, a cheerful, contented disposition, and temperate habits are, with rare exceptions found indispensable. Now these pre-suppose a harmonious development of the whole body, and particularly of all parts of the brain. For it is impossible, we believe, to obtain the qualities here mentioned in a high degree without these two conditions. And the nearer this development approaches that standard of organization upon which is based the great law of longevity, the greater will be not only the aggregate amount of health, but the longer the duration of human life. This statement will be found abundantly verified in the history and character of persons who have reached a great age.—Dr. Nathan Allen.—*The Sanitarian for February.*

---

*The New Local Anæsthetic.*—The observation of Horwath, of Kiew, that absolute alcohol at a temperature of 20° Fahr. is a most efficient local anæsthetic, deserves to be remembered and acted upon. He finds it far superior to cold ether, or ice, or the spray of volatile substances.—*Med. and Surg. Reporter.*

*Microscopic Terrors.*—This is the age of detectives in medicine. The best minds are pursuing the physical causes of disease, with the microscope, with chemistry, with imagination. Disease is no longer dynamic but material. Its seeds float in the air, and abound in water, in milk, in food. When one takes a drink of water the chances are that he swallows a myriad of living organisms, vegetabl. and animal, which will carry into his blood the germs of typhoid, of tubercle, of cholera. Milk may be fraught with the poison of typhoid fever, and may scatter the pestilence broadcast. It may conceal the seeds of cholera and plant a great harvest of that scourge. So we are taught. More than that, we are now assured that milk can propagate tubercle. A French scientist has demonstrated that calves fed on substances with which tuberculous matter is mixed, will become tuberculosis, and of course that the milk of tuberculous women will do the same. And then we can not breathe the air with safety, for it may be filled with the germs of palmella, which will enter the blood and grow into an ague-fit. Pork and beef are dangerous from the trichina, and tenia is smuggled into the stomach with vegetables and spring water. We live in a dangerous world, and what with the multiplication and improvements of binoculars the dangers increase every day. It is to be hoped our microscopists will give us a bill of fare, informing us what we may eat and drink, if indeed there is anything salubrious.—*Pacific Med. and Surg. Journal.*

---

*Methylene Ether as an Anæsthetic.*—By the introduction of this new anæsthetic, Mr. Lawson Tait feels sure that the days of chloroform as an anæsthetic, for any but obstetric operations, are numbered. The new substance has the following advantages over chloroform: Its action is much more rapid, and is entirely free from the muscular and cerebral excitement often seen in the use of chloroform; the quantity used is less, the sickness after its use is more exceptional, the recovery from the anæsthesia being extremely rapid and complete. Over sulphuric ether it has the advantage that it is very pleasant to take, and that a tenth or twelfth of the quantity is sufficient. He has used it about thirty times, and only in one instance was there any sickness, and in that case the lady had just before the examination, been partaking freely of underdone mutton.

Mr. Tait says he shall use no other anæsthetic for surgical work until he obtains some disastrous result, a misfortune that at present seems more unlikely than by the use of either ordinary ether or chloroform. He adds that its use is more economical than that of either ether or chloroform.—*British Med-Journal.*

*The Human Body Compared to a Machine.*—In the promotion of health and longevity, too much stress cannot be attached to the importance of preserving this harmony or balance of organization. In some respects, the human body may be compared to a perfect machine, made up of many complicated parts. How different the working or running of such a machine from that of one imperfectly constructed and unequally balanced in all its parts! The one seldom needs repairs, the other frequently. The one will last as it were for an age; the other becomes almost useless in a short time.

It is so in reference to the human system. Whenever a certain organ or class of organs becomes relatively too large or too small, causing a want of balance or harmony in their action, there must be in the very nature of the case far greater liability to disease. Accordingly, it is in persons possessing this imperfect, ill-balanced organization, that we find not only the greatest amount of sickness, but that which is most obstinate and fatal. How often it happens that some slight derangement or trifling weakness operates as the entering wedge to the most serious diseases! It is the weak spot caused by inheritance, or developed by exposure, where disease finds its germ or starting point. through all other parts of the system are in a perfectly sound condition; and not unfrequently life is terminated by a single organ, or even some part of it giving out, when all the other organs might have performed their healthy functions for many years.—Dr. Nathan Allen.—*The Sanitarian for February.*

---

*Bad Effects of Thumb-Sucking.*—A year or two ago a writer, whose name escapes us just now, showed that the habit of thumb-sucking in children leads indirectly to mental feebleness. Quite recently Dr. Horace Dobell writes to an English exchange:—"I have observed that a peculiar and rather common deformity of the chest is caused by the habit of sucking the thumb in infancy and early childhood. The weight of the arm on the thorax of the child during sleep, produces depression of the ribs in the line occupied by the arm when the thumb is placed in the mouth. As this is a very important effect of 'thumb-sucking,' never hitherto pointed out, I think it desirable to place this note on record, for the benefit of other observers."—*Med. and Surg. Reporter.*

---

*Sugar and Magnesia an Antidote to Arsenic.*—The *Mouvement Medical* relates various experiments conducted by Mr. Carl, with the result of showing that sugar mixed with magnesia may serve as an antidote in cases of poisoning by arsenious acid, in which cases, too, the internal use of the hydrated magnesia is most valuable.

*Nervous or Sick Headache.*—In those forms of sick headache which are preceded by disturbed vision, or other signs recognizable by the patient as preceding an attack, the patient should lie down with the head as low as possible, and if the glimmering be on the right or left of the field of vision, he should lie on the opposite side. He should at the same time take some powerful diffusible stimulant. By this means the defective supply of blood to some portion of brain, which is the real disease, is counteracted. There is always a loss of tone about the cerebro-spinal system in cases of this kind, and of course all measures calculated to improve the general health should be adopted. If the attack is followed or preceded by great mental depression, nothing acts like half a drachm or a drachm of the ammoniated tincture of valerian. A remedy which is often given with great advantage during a severe attack is bromide of potassium in doses of 5, 10, to 30 grains, combined with 30 or 40 minims of salvolatile. If the attacks have been very frequent, or if there be any scrofulous tendency, the iodide of iron may be given in the following form: ℞ Ferri et ammon. cit., gr. v.; potassii iodidi gr. ij.; aquæ ℥j., and, according to circumstances, 15 to 20 minims of tincture of henbane, or 20 or 30 minims of aromatic spirits of ammonia may be added. If the stomach is irritable this may be given in the effervescing form. In other cases citrate of iron with ammonia and strychnine may be given with great success.—*Braithwaite.*

---

*The "Blood Cure."*—We have had the "milk cure," and the "water cure," and now we are offered the "blood cure." Invalids in Boston have commenced to patronize the Butchers' Abbatoir, in Brighton, for medical treatment, simply drinking a half tumblerfull of warm blood twice a day. This course, one gentleman, Mr. C. H. Stickney, has followed for ten weeks, and during that time gained ten pounds in weight. Another, a consumptive, so feeble that it was with difficulty he could get to this abbattoir, is now able to handle an axe skillfully enough to "knock down a bullock." A lady sick six years, stricken with paralysis, is improving wonderfully. A gratifying feature of this cure is that it is "without money and without price."—*Med. and Sury. Reporter.*

---

*The Diagnosis of Lipomata.*—An excellent suggestion is made in a French journal. A character peculiar to lipomata resides in the property belonging to all fatty tumors, of hardening under the action of cold. When, after the use of ice or the ether spray, in the case of a doubtful tumor, the growth is felt to become harder, the presumption is that the case is one of lipomata.—*Med. and Surg. Reporter.*

*Important Point in the Treatment of Frozen Limbs.*—It is well known that after a limb has been frozen by exposure, no matter what care is exerted in restoring the circulation *gradually*, there remains a capillary stasis, a blue color, and great danger of violent inflammation and gangrene.

Dr. Bergmann, of Dorpat, recommends and has successfully practiced *vertical suspension of the limbs*, which promptly disperses the venous stasis of the period of reaction with its attendant dangers. If, as is most common, the feet and legs have been frozen, they should be held considerably higher than the trunk, so as to facilitate the reflux of blood to the heart.—*Med. and Surg. Reporter.*

---

*Laws of Health.*—With the increased knowledge and observance of the laws of health, many individuals have not only prolonged their own lives, but the average duration of human life, within forty or fifty years, has considerably advanced. But physiology in its practical applications is yet in its infancy. When its principles become so generally understood and appreciated as to be practically applied through-out the community, in every family, and by every individual, then will be found a great diminution of disease, as well as of early mortality.—Dr. Nathan Allen.—*The Sanitarian for February.*

---

*Ether Glue.*—An excellent liquid glue is made by dissolving glue in nitric ether. The ether will only dissolve a certain amount of glue, consequently the solution cannot be made too thick. The glue thus made is about the consistency of molasses, and is doubly as tenacious as that made with hot water. If a few bits of India-rubber, cut into scraps the size of buck-shot, be added, and the solution be allowed to stand a few days, being stirred frequently, it will be all the better, and will resist the dampness twice as well as glue made with water.—*Druggists Circular.*

---

*Fuschin as an Antiseptic.*—This anilin preparation is said by Laujonois, in the *Comptes Rendus*, to be a remarkably powerful antiseptic. One per cent, added to a solution of gelatine, not only preserved it, but a piece of fresh meat wrapped in paper soaked in this medicated gelatine solution kept for months without a sign of putrefaction. One forty thousandth part added to urine, prevents it perfectly from decomposing. Such experiments merits careful repetition.—*Med. and Surg. Reporter.*

*Women Dentists in Egypt.*—Prof. Edward Warren writes from Cairo, in Egypt, to a friend in Baltimore, that there is "a good opportunity for women dentists in Egypt, as in the East, women are forbidden to consult with men." There are three or four English women practicing dentistry in Cairo already, according to Dr. Warren's letter. In all those Eastern countries there seems to be a wide field of usefulness and profit for women doctors and dentists.—*Med. and Surg. Reporter.*

---

*Chloroform in Dentistry.*—At the late meeting of the Society, the following resolution was almost unanimously adopted :—

"*Resolved,* That, in the opinion of the Massachusetts Dental Society, the use of chloroform in dental operations is inadvisable."

---

*Persecution of Male Students.*—The *Medicinische Central Zeitung,* Jan. 14, says that 30 young women, chiefly Russians and Poles, have inscribed in the Zurich University. They rudely sieze the best seats, and crowd the poor young men at the clinics; they even smoke to such an extent that the professors complain that it makes them cough. The poor persecuted male students have therefore petitioned the Senate to stop such improprieties. These afflicted youths have our warmest sympathy. —*Exchange.*

---

*Treatment of Burns and Scalds.*—Dr. de Breyne highly recommends the following treatment in *L' Union Pharmaceutique;* Hydrate of lime (newly precipitated), forty-five grains; glycerine, five ounces; chloric ether, forty-five drops. It makes up a transparent, colourless liquid, with an agreeable odour, and an alkaline reaction, according to the dose of hydrate of lime. It calms the pain, and prevents or abates inflammation.—*Lancet.*

---

*Lime-water in Stings of Bees and Wasps.*—M. Dauverne states (*L' Union Med.,* Oct. 25) as the result of numerous trials, that the pain and suffering caused by these may be immediately assuaged by the application of lime-water—a remedy which may always be prepared at once by the aid of a little quicklime and a glass of water.—*Med. Times and Gaz.*

